# Sulphate of Quinine in the Local Treatment of Ulcers

**Published:** 1894-01

**Authors:** 


SULPHATE OF QUININE IN THE LOCAL TREATMENT OF ULCERS. (*Giornale di farmacia, di chimica e di scienze affini*, Turin, May, 1893.) By Alfordi.

The author has noticed that infectious ulcers which seem to be rebellious to all kinds of treatment assume at once a healthy appearance and heal quicker when bathed with a solution of one per cent. of sulphate of quinine than when washed with a solution of corrosive sublimate or dusted with iodoform. Regarding simple ulcers, the healing action of the sulphate of quinine is still more rapid.—*Ex.*

